# Ultrasound-Guided vs. Fluoroscopy-Guided Interventions for Back Pain Management: A Systematic Review and Meta-Analysis of Randomized Controlled Trials

**DOI:** 10.3390/diagnostics13223474

**Published:** 2023-11-18

**Authors:** Dmitriy Viderman, Mina Aubakirova, Anuar Aryngazin, Dinara Yessimova, Dastan Kaldybayev, Ramil Tankacheyev, Yerkin G. Abdildin

**Affiliations:** 1 Department of Surgery, Section of Anesthesiology, Intensive Care, and Pain Medicine, Nazarbayev University School of Medicine (NUSOM), Astana 020000, Kazakhstan; mina.aubakirova@nu.edu.kz (M.A.); or d.yessimova@campus.tu-berlin.de (D.Y.); 2 Department of Mechanical and Aerospace Engineering, School of Engineering and Digital Sciences, Nazarbayev University, Astana 010000, Kazakhstan; anuar.aryngazin@nu.edu.kz (A.A.); dastan.kaldybayev@nu.edu.kz (D.K.); yerkin.abdildin@nu.edu.kz (Y.G.A.); 3 Department of Health Care Management, Faculty of Economics & Management, Technische Universität Berlin, 13355 Berlin, Germany; 4 Department of Pain Medicine, National Neurosurgery Center, Astana 010000, Kazakhstan; ramiltankacheyev@gmail.com

**Keywords:** back pain management, ultrasound, fluoroscopy, injections, pain intensity, postoperative complications

## Abstract

The objective of this study was to compare the outcomes of the ultrasound- and fluoroscopy-guided techniques in the management of back pain. Using PubMed, Scopus, and the Cochrane Library, we searched randomized controlled trials (RCTs) published before May 2023, which reported relevant data on the topic. The effectiveness of the ultrasound-guided (US-guided) and fluoroscopy-guided (FL-guided) approaches for back pain management was compared in terms of postoperative pain intensity, postoperative functional outcomes, and postoperative complications. Subgroup analyses were conducted for different postoperative periods. Eight studies were included in the analysis. There was no significant difference in post-procedural pain relief at one week, two weeks, one month, two months, and three months between the US-guided and FL-guided interventions for back pain management (SMD with 95% CI is −0.01 [−0.11, 0.10]), *p* = 0.91, I^2^ = 0%). In terms of the postoperative functional outcomes assessed by the “Oswestry Disability Index” (ODI) functionality score, the model tends to favor the FL-guided injections over the US-guided injections (SMD with 95% CI: 0.13 [−0.00, 0.25], *p* = 0.05, I^2^ = 0). Finally, the US-guided and FL-guided injections did not show significantly different results in terms of postoperative complications (RR with 95% CI is 0.99 [0.49, 1.99], *p* = 0.97, I^2^ = 0). The subgroup analysis also did not demonstrate differences between the US-guided and FL-guided techniques in the following outcomes: vasovagal reaction, transient headache, and facial flushing. There was no significant difference between the US-guided and FL-guided injections for treating back pain in terms of postoperative pain intensity and complications. Still, the model tends to favor the FL-guided injections over the US-guided injections in terms of functionality.

## 1. Introduction

Back pain is extremely common in the adult population, and it is one of the most frequent causes for patients to seek medical care [1,2,3]. The conservative medical treatments for addressing back pain consist of attempting oral medication and manual and exercise therapies. Interventional pain management modalities have been used for many years for the management of back pain [1,2,3]. The conservative medical treatments for addressing back pain consist of attempting oral medication and manual and exercise therapies. Commonly performed interventional procedures for back pain management include epidural steroid injections, caudal steroid injections, selective nerve root blocks, facet joints, and/or medial branch blocks. Such procedures provide a relatively long-term pain-alleviating effect, with the most advantageous outcome typically observed several weeks after the procedure [4]. Nevertheless, there may be differences in maximal efficacy and duration of action. Therefore, there are no uniform follow-up timings. The literature suggests that control visits are usually scheduled for the 1 week, 1 month, 2 months, and 3 months following the procedure. Sometimes, follow-ups occur on week 2, and in case of unsatisfactory anesthetic effect, the procedure may be repeated 3–4 weeks after the initial intervention. Although blind caudal epidural injections have a success rate of 74–90% [4], fluoroscopy-guided (FL-guided) techniques are more commonly used as they provide more accurate visualization and needle placement, resulting in subsequently accurate administration of the injectate and better outcomes [1,2,3,5]. 

The success rate of fluoroscopy-guided injections is generally higher compared to blind techniques and can vary depending on several factors, including individual patient factors, underlying cause and mechanism of pain, location of pain generators, specific injection technique, skill, and experience of specialists performing this procedure [5]. However, this technique has several negative effects, including radiation exposure, the contrast agent utilized to confirm appropriate drug deposition that has been associated with various side effects, including nausea and vomiting, hives, bronchospastic reaction, urticaria, hypotension, tachycardia, and anaphylactic reaction [2,3]. US-guided techniques have recently started gaining popularity among interventional pain physicians [6]. Ultrasound has proven to provide real-time visualizations of anatomical structures with less cost and reliable imaging for finding successful injection sites [7]. The success rate of US-guided low back pain interventions has been reported at 85–100% [8,9,10]. Previous studies compared the effects of caudal epidural steroid injections under the guidance of FL- and US-guided imaging by assessing post-procedural outcomes using the following scales: “Visual Analogue Scale” (VAS), “Visual Numeric Scale” (VNS), “Numeric Rating Scale” (NRS), “postoperative Oswestry Disability Index” (ODI), “Neck Disability Index” (NDI) functionality scores, and frequency of postoperative complications among patients [1,2,3,10]. Previous observational and randomized controlled trials reported mixed results regarding the efficacy and safety of these methods, but many of them did not find any significant difference. The objective of this SR&MA was to compare the US- and FL-guided interventions in reducing back pain intensity, functional outcomes, and complications.

## 2. Materials and Methods

### 2.1. Protocol

**Figure 1:**
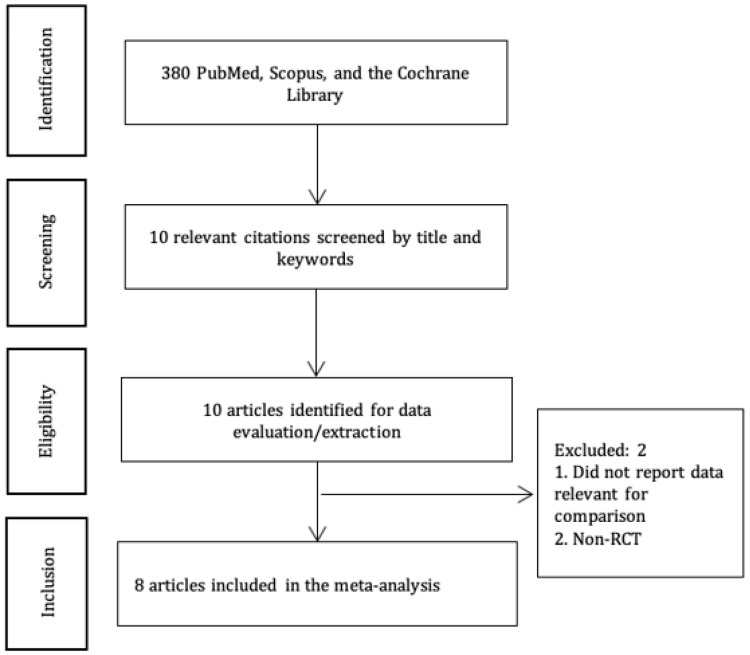
PRISMA diagram.

The current SR&MA was conducted following the PRISMA guidelines [11]. The protocol was publicly registered in the Open Science Framework at https://doi.org/10.17605/OSF.IO/CBQ82. We searched for RCTs, which studied the effects of the US-guided and FL-guided injections for back pain management, published before May 2023 in Scopus, PubMed, and the Cochrane Library (Figure 1). The following search terms and/or their combinations were used: “fluoroscopy”, “fluoroscopic guidance”, “fluoroscopy-guided”, “ultrasound”, “ultrasound-guided”, “sonography”, “diagnostic imaging”, “diagnostic”, “imaging”, “ultrasonography”, “ultrasonics”, “ultrasounds”, “back pain”, “low back pain”, “radicular pain”, and “epidural injections”.

Two authors conducted the screening. First, the articles were screened based on titles. Then, abstracts were screened based on inclusion/exclusion criteria. Finally, full texts were screened, and those articles that reported outcomes of interest for this study were included in the meta-analysis. In case of disagreements, a third author was consulted.

We considered the following criteria for inclusion: (a) study design: “randomized controlled trials” (RCTs); (b) age: 18 years and older; and (c) procedures: interventions for back pain management performed either under US and/or fluoroscopic guidance. We considered studies published in the English language. 

We excluded studies if they included (a) pediatric patients; (b) study designs other than RCT; or (c) poorly described methodology or inadequately reported findings. 

### 2.2. Outcomes

The primary outcome of our meta-analysis is post-procedural pain intensity. The secondary outcomes were functional outcomes (ODI/NDI) and postprocedural complications.

### 2.3. Data Extraction and Biostatistics Methods

Data extraction disputes were resolved via discussion among the authors. In case of further disagreements, an author not involved in data extraction was consulted. We extracted the following data from RCTs in the data table (Table 1): 1st author, reference, country, study design and goals, patient age, study groups, number of patients, and interventions. If more than one study reported the outcomes of interest, we added them for data synthesis. Statistical methods were used for data conversions [12,13]. First, the data were extracted into an Excel file, and then a meta-analysis was conducted using the random effects model in RevMan 5.4 software, “the Cochrane Collaboration”. In data synthesis, the standardized mean difference was used for the outcomes of pain intensity and ODI/NDI, and the risk ratio was used for the incidence of postprocedural complications. The results were presented with forest plots, where green/blue squares with lines represent sample means with 95% CIs in a single study, whereas black diamonds represent the same for a group of studies (i.e. the overall result). I^2^ statistic was used to estimate the heterogeneity.

### 2.4. Methodological Assessment

We used the Cochrane Risk of Bias tool 2 [14] to check the methodological quality of studies. Each study was assessed in terms of bias in the randomization process, deviations from the intended interventions, missing outcome data, measurement of the outcome, and selection of the reported results. For each of these domains, the risk of bias was graded as “high”, “low”, or “some concerns”. For example, for the randomization process, a “low” risk of bias implied appropriate allocation sequence generation and concealment that was well described and resulted in comparable groups at baseline. Based on the results for each domain, an overall assessment of bias for the study was drawn. If one to two domains were rated as having “some concerns”, the overall assessment for the study was “some concerns”. If three or more domains were rated as “some concerns” or at least one as “high risk”, the study was given an overall rate of “high” risk of bias. The main outcomes were assessed with GRADE [15] and presented in the Summary of Findings table. For each outcome, the certainty of evidence was evaluated based on the risk of bias, inconsistency, indirectness, and imprecision. Each of these criteria was rated as “not serious”, “serious”, or “very serious”. “Serious” or “very serious” concerns about the certainty of evidence downgraded the certainty of evidence from high to very low. Two authors conducted the quality assessment. Risk of bias assessed the randomization and blinding processes, incomplete accounting of patients or events, selective outcome reporting, and other potential sources of bias. Inconsistency took into account unexplained heterogeneity, the width of variance of the point estimates, and the overlapping of the confidence intervals. Indirectness checked whether the evidence was drawn from populations, interventions, outcome measures, or comparisons different from the ones of interest for a given outcome. Imprecision considered the number of patients and the width of the confidence interval. Upgrading was possible when the pooled effect size was large, when there was a dose–response effect, or when the effect could have been a result of residual confounding.

## 3. Results

We initially identified 380 articles that matched our search criteria (Figure 1). Eight articles [2,3,6,7,16,17,18,19] with 642 patients (US-guided group—321 and FL-guided group—321) were selected for a meta-analysis (Table 1). 

**Table 1:** Study characteristics. Abbreviations: TFEI, transforaminal epidural injection; US, ultrasound; FL, fluoroscopy; SNRB, selective nerve root block; CESI, caudal epidural steroid injections; ESI, epidural steroid injections; SIJI, sacroiliac joint injections; SIJ, sacroiliac joint; St Dev, standard deviation; N, number; RCT, randomized controlled study.

Author, Citation	Country	Study Design	Study Goals	Age Mean (St Dev)	N of Patients: Total (Intervention/Control)	Patient Characteristics	Injected Agents	Study Conclusion
Akkaya et al., 2017 [6]	Turkey	RCT	To compare outcomes of FL- or US-guided CESI in post-laminectomy patients	48.55 (10.66), 45.26 (9.83)	30: (15/15)	“Patients who had undergone L4-5 or L5-S1 hemilaminectomy within the last 1 year”	“2.5% bupivacaine + dexamethasone 8 mg”	Comparable effectiveness; US more comfortable
Evansa et al., 2015 [3]	Latvia	RCT	To assess the technical feasibility of US- and FL-based methods in ESI	69.2 (10.3), 69.1 (10.2)	112: (56/56)	“Spinal canal stenosis, disc herniation, spondylolisthesis”	“Corticosteroid (methylprednisolone acetate 80 mg) + 1% lidocaine 4 mL”	Comparable analgesic effect and time to perform
Jee et al., 2013 [2]	Republic of Korea	RCT	To compare short-term analgesic effect, functional enhancements, and safety between US-guided SNRB and FL-guided TFEI in the lower cervical spine	57.76 (9.56), 56.69 (9.32)	110: (55/55)	“Patients with posterior neck and radicular pain”	“1 mL of 1% lidocaine, then 2 mL dexamethasone (10 mg) and 1 mL 0.5% lidocaine”	Comparable effectiveness in reducing pain
Yang et al., 2016 [7]	China	RCT	To assess the precision, impact on pain relief, and safety of US-guided lumbar TFEI	57 (10), 58 (9)	80: (40/40)	“Back pain associated with lower limb radiation pain, herniated disk or spinal stenosis”	“1% lidocaine, 4 mL of 0.5% lidocaine + 1 mL of diprospan”	Feasibility and safety of US, success rate of 85%
Hazra et al., 2016 [16]	India	RCT	To evaluate the time taken for precise needle positioning and analyze the clinical effectiveness of FL and US guidance in CESI in chronic low back pain	44.48 (6.48), 41.88 (8.05)	50: (25/25)	“Chronic low back pain with unilateral or bilateral radiculopathy of more than 3 months duration, not responding to conventional therapy”	“2 mL preservative-free lignocaine (1%) pre-procedure, methylprednisolone 40 mg diluted in 10 mL normal saline”	Comparable pain-sparing effect, better visualization in FL
Soneji et al., 2016 [17]	Canada	RCT	To investigate the differences in accuracy and effectiveness between US- and FL-guided SIJI	50.90 (12.77), 46.85 (11.51)	40: (20/20)	“Patients with chronic back pain secondary to SIJ arthritis”	“40 mg of methylprednisolone acetate diluted in 3 mL of bupivacaine 0.25% with epinephrine 1:200,000 (total 4 mL injectate)”Fl: “radio-opaque contrast 0.5 mLs followed by fluoroscopy imaging”	Comparable accuracy, efficacy, and overall patient satisfaction
Park et al., 2013 [18]	Republic of Korea	RCT	To assess the immediate benefits of US-guided CESI with FL-guided ESI for unilateral radicular pain in the lower lumbar spine	57.27 (10.11), 58.47 (9.22)	110: (55/55)	“Patients with unilateral lumbar radicular pain”	“Nonionic contrast medium: 5 mL (Omnipaque 300) + 15 mL (13.0 mL of 0.5% lidocaine + 2 mL of dexamethasone 10 mg)”	Comparable analgesic effect, functional improvement, and patient satisfaction
Jee et al., 2014 [19]	Republic of Korea	RCT	To examine the efficacy and outcomes of US-guided and FL-guided SIJI in noninflammatory SIJ dysfunction	60.98 (8.58), 60.69 (8.02)	110: (55/55)	“Chronic low back pain (>3 mo) without radiculopathy”	“0.5 mL nonionic contrast media (Omnipaque 300a) + 2 mL (1.0 mL 0.5% lidocaine + dexamethasone 10 mg)”	Comparable efficacy

### 3.1. Qualitative Description of the Studies

The included studies were published between 2012 and 2018. The studies presented the results of pain measurements before the injection, at one and two weeks, and one, two, and three months post-operation, while ODI/NDI functionality scores were measured at baseline, two weeks, and one and three months. Immediate post-injection complications such as vasovagal reaction, transient headache, and facial flushing were the common reactions encountered by a minority of patients. 

#### 3.1.1. “US-Guided Selective Nerve Root Block” and “fluoroscopy-Guided Transforaminal Block” for the Treatment of Radicular Pain in the Lower Cervical Spine

We found one publication that studied this procedure [2]. The “US-guided intervention selective nerve root block” was shown to facilitate the identification of vessels and avoid injury to the vessels. It is important to note that injury to the vessels is considered the leading cause of complications in “cervical transforaminal injections”. Therefore, the absence of radiation, safety in terms of avoiding vessel injury, and real-time imaging could make US guidance a preferred method.

#### 3.1.2. US and “FL-Guided Epidural Steroid Injections” in Patients with Degenerative Spinal Diseases

It was found that “US-guided epidural steroid injections” demonstrated a similar analgesic effect compared with “FL-guided injections” [3]. There was no difference between the two techniques in terms of time to perform interventions. The highest pain-relieving effect was observed 4 weeks post-procedure. At 3 months, the effect lowered but was still present.

#### 3.1.3. US and “FL-Guided Caudal Epidural Steroid Injection” in Unilateral and Bilateral Radiculopathy

The authors found significant improvements in pain and disability index within both groups compared to the baseline scores and no difference between the groups [16]. It was also shown that the US might be a safe alternative for the FL for placing the needle in the caudal epidural space for the procedure. Moreover, correct needle placement was faster under US guidance. The authors, however, stress FL’s superiority in terms of providing a clearer visual of the needle tip and the patterns of the epidurogram [16]. 

#### 3.1.4. US and “FL-Guided Caudal Steroid Injections” for Degenerative Spinal Diseases

One study assessed US and “FL-guided caudal steroid injection” for the management of post-laminectomy pain [6]. “Caudal epidural steroid injection” was reported to be a safe and easy analgesia method for post-laminectomy patients. It is one of the most commonly used interventions in chronic pain treatment after low back surgery. Unfortunately, if this intervention is performed under the classical landmark technique, it can lead to various complications, including dural puncture, intraosseous injection, infection, and hemorrhage. “US-guided interventions” might be more advantageous compared with “FL-guided interventions” since they showed better patient satisfaction and reduced the duration of the intervention. US-guided injection is a safe modality to locate the sacral hiatus and perform the needle placement [6].

#### 3.1.5. US-Guided and “Fluoroscopy-Controlled Lumbar Transforaminal Epidural Injections”

There was no difference in pain reduction between the US and FL groups. The success rate of the US-guided interventions was 85%. The procedural time was shorter in the US than in the FL group, and the radiation dosage in the US group was lower than in the FL group. There were no serious complications reported in any of the patients in either group. Therefore, US guidance might be a safe, feasible method that requires less time and is associated with less radiation to reach the same results as the FL-guided method [7].

#### 3.1.6. US-Guided vs. “FL-Guided Caudal Epidural Steroid Injection” in Unilateral Lower Lumbar Radicular Pain

It was shown that there were no significant improvements in pain and ODI scores at 2 weeks and 3 months in both groups, and the intergroup difference in scores [18]. The needle repositioning rate was also similar, 13.3% for the FL group and 15% for the US group. A significantly higher number of injections in the opposite side of the lesion occurred in the US group [18]. 

#### 3.1.7. “Fluoroscopic Guidance” vs. “Ultrasound Guidance for Sacroiliac Joint (SIJ) Injection” in Chronic Low Back Pain

The study conducted by Soneji did not find any notable differences in accuracy, efficacy, or patient satisfaction between these two image-guided techniques for SIJ injections [17].

### 3.2. A Meta-Analysis of All Studies Combined

#### 3.2.1. Pain Intensity (VAS, VNS, NRS Scale)

**Figure 2:**
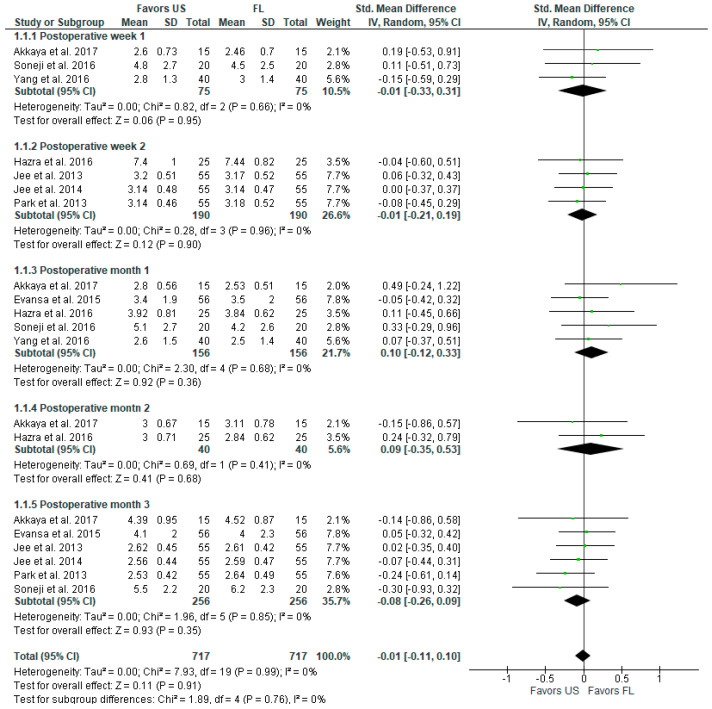
Pain intensity (VAS, VNS, NRS scale) [2,3,6,7,16,17,18,19].

Pain intensity is shown in the forest plot below (Figure 2). The model does not favor US or FL at postoperative weeks 1 and 2 and months 1, 2, and 3. The overall pain intensity at all the periods combined is not statistically significant at SMD with 95% CI −0.01 [−0.11, 0.10]), *p* = 0.91, I^2^ = 0%.

#### 3.2.2. Postoperative Functional Outcomes and Disability (ODI Index)

**Figure 3:**
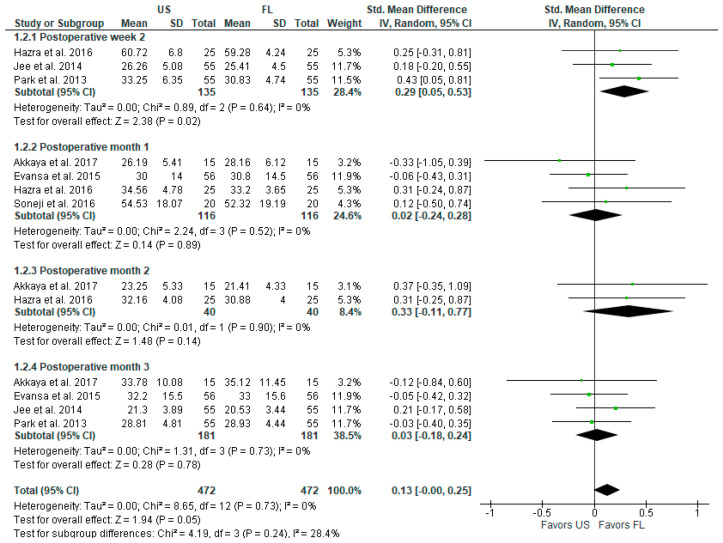
Postoperative functional outcomes (ODI index) [3,6,16,17,18,19].

FL appears to be slightly superior to the US in terms of ODI at 2 weeks postoperatively at SMD with 95% CI 0.29 [0.05, 0.53], *p* = 0.02, I^2^ = 0% (Figure 3). Despite being statistically significant, this result is not clinically meaningful. Additionally, there were no statistically significant differences in ODI at 1, 2, and 3 months postoperatively. The overall result of the model tends to favor the FL-guided injections over the US-guided injections: SMD with 95% CI 0.13 [−0.00, 0.25], *p* = 0.05, I^2^ = 0%.

#### 3.2.3. Postoperative Complications

**Figure 4:**
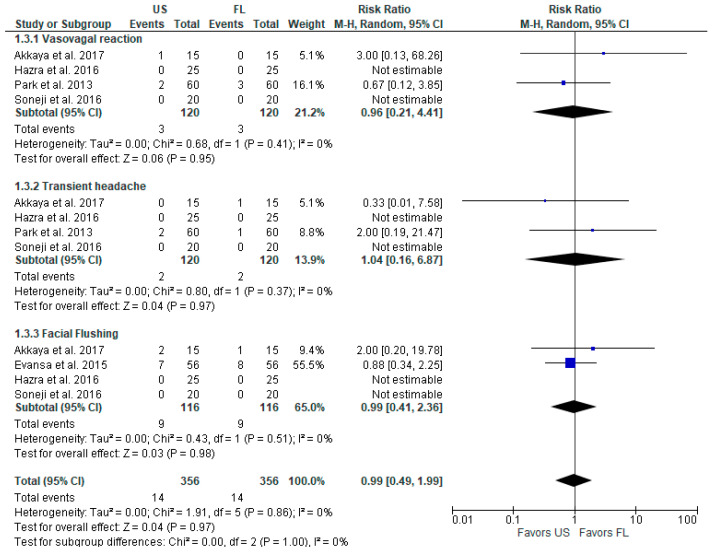
Postoperative complications [3,6,16,17,18].

Both approaches appear to have comparable safety in terms of postoperative vasovagal reaction, transient headache, and facial flushing (Figure 4). Combined together as postoperative complications, the overall model does not favor either US-guided or FL-guided injections at a risk ratio with a 95% CI of 0.99 [0.49, 1.99], *p* = 0.97, I^2^ = 0%. We should note that two studies (Hazra (2016) [16] and Soneji (2016) [17]) did not observe any postoperative adverse events.

### 3.3. Assessment of Methodological Quality

Given the nature of the procedure, a certain degree of risk of bias was present due to the lack of double blinding. This has seriously affected the methodological quality of the studies. Based on the Cochrane risk of bias assessment, three studies had “some concerns” about the risk of bias, and four had a “high” risk of bias (Table 2). Five outcomes were assessed for quality (Table 3). Of them, one was of moderate quality, and four were of low quality. Detailed assessment of the outcomes is provided in the Evidence profile table in Supplemental Materials.

## 4. Discussion

In this SR&MA, we included eight RCTs, which compared ultrasound-guided and fluoroscopy-guided techniques for back pain management. Using ultrasound guidance alongside fluoroscopic confirmation could be a viable option for visual guidance for various interventions in back pain management [2,3,6,7,16,17,18,19].

There were no significant differences found in the reduction in back pain at one week, one month, and three months following the interventions. Regarding postoperative functional outcomes assessed by the ODI and NDI, there was no difference between the two groups at one month, and three months after intervention. The rate of postoperative complications also did not differ significantly between US-guided and FL-guided techniques. Subgroup analysis that was performed for various outcomes (vasovagal reaction, transient headache, facial flushing) did not differ significantly between US-guided and FL-guided injections [2,3,6,7,16,17,18,19].

A recent meta-analysis compared US-guided injections (intervention group) with fluoroscopy- or computer tomography (CT)-guided injections (control group) in the spinal nerves [20]. Similar to our results, the study found no difference in pain scores between the US- and FL/CT-guided interventions for low back pain. Likewise, the authors concluded that there was “little to no difference” in ODI/NDI scores between the US- and FL-/CT-guided injections, similar to our results. The authors then conducted a sub-group analysis, separating the cervical group from the lumbar one. In this sub-group analysis, both pain and functional disability scores remained comparable between the US and the FL/CT groups. Regarding adverse events, the authors suggest that both major and minor complications seem to be less likely to occur in the US-guided intervention group. Moreover, US-guided injections were associated with a lower duration of the procedure [20].

Observational studies showed similar results. Thus, a study comparing “US-guided selective nerve root block”, “FL-guided interlaminar epidural block”, and “FL-guided transforaminal epidural block” for the management of radicular pain in the lower cervical spine demonstrated that although both the VNS and “neck disability index” improved within the groups at 1, 3, and 6 months in all groups, there were no differences between the groups. Moreover, the treatment success rate at all time points was not significantly different between the groups. US-guided interventions required a shorter duration and resulted in similar pain reduction and functional improvement. Thus, a US-guided technique might be considered for epidural steroid injection [21].

Similar results were yielded by another retrospective observational study of 54 patients with facet syndrome [22]. One month post-procedure, the pain scores improved within the group but did not differ between the US- and FL-guided groups. There were no major complications in either group. The researchers suggest using US guidance as a safe and equally effective substitute for FL-guided injections to avoid excessive irradiation.

Another observational study comparing US- and FL-guided caudal epidural steroid injection for the management of unilateral lower lumbar radicular pain also yielded similar results [23]. The study showed that the Oswestry Disability Index and verbal numeric scale scores improved at 3, 6, and 12 months in both groups. There were no statistical differences in verbal numeric scale scores and Oswestry Disability Index and the proportion of patients with successful treatment between the groups. Therefore, US-guided techniques might be considered in the management of lower lumbar radicular pain.

Ultrasound could be used with fluoroscopic confirmation for guiding sacroiliac joint injections in patients with SIJ arthritis. Both FL-guided and US-guided approaches for different methods of SIJ innervation are well described in a review article [24]. Even if fluoroscopic guidance is used for confirmation, the radiation exposure could be significantly reduced. There were no notable differences in accuracy, efficacy, or patient satisfaction between these two image-guided techniques for SIJ injections [19]. The rates of intra-articular injection during US-guided SIJ injections have shown significant variability in prior studies. Three previous studies have reported higher rates of intra-articular injection, ranging from 76.7% to 87.3% [25,26,27]. However, it is worth noting that the first two studies included relatively younger patients with inflammatory SIJ arthropathy and excluded patients with osteoarthritis of the SIJ. In contrast, our study included patients with an average age of 48.9 years who were more likely to have osteoarthritic changes. Accessing the intra-articular portion of the joint becomes more challenging in osteoarthritic conditions compared to inflammatory pathologies like rheumatoid arthritis or ankylosing spondylitis. Furthermore, a meta-analysis found that the risk difference of incorrect needle placement of US-guided injections was 13% for facet joint injections, as confirmed by CT, and 11% for lumbar medial branch blocks, as confirmed by FL guidance [28]. The quality of evidence, however, was from very low to low. Therefore, differences in intra-articular injection rates may be attributed to patient characteristics and pathologies studied.

Hartung et al. found that using ultrasound for sacroiliac joint (SIJ) arthropathy resulted in 40% of injections being administered intraarticularly [29]. Remarkably, both intra- and periarticular injections provided similar pain relief benefits. Other studies have also suggested that periarticular injections could have comparable outcomes to intraarticular injections. In Hartung’s study, the reduction in pain scores 1 month after the procedure (25% to 33%) was similar to that reported in the study by Soneji [17,27]. Overall, the success rate of intraarticular injections using the US for SIJ injections falls within the wide range reported in the literature, and the results may vary depending on the characteristics of the study populations.

Given the absence of radiation exposure, lower cost, and convenience, US-guided techniques might be preferred for patients, doctors, and healthcare in general. The current guidelines for interventional management of chronic back pain recommend using fluoroscopic guidance. A qualitative review of studies on intradiscal injections for low back pain management revealed that almost 62% of the studies on the topic had performed the procedure under FL guidance (with or without comparators), and another 8.8% combine the FL and CT approaches [30]. Another narrative review described US, FL, and CT visualization in chronic pain interventions [31]. Despite the firm adherence to the guidelines on the use of fluoroscopy-guided procedures, adverse events have been reported. The complications may occur if the needle penetrates vitally important vessels, such as the anterior spinal artery [6,7,18]. Ultrasound guidance allows better visualization of vessels, nerves, soft tissue, and the spread of the anesthetic solution around the nerve. Therefore, it might provide an operator with better identification of vulnerable anatomical structures, while the value of fluoroscopy in identifying such anatomical structures is limited [2]. The advantages of the US are that it is radiation-free, easy to use, and it can offer continuous real-time needle guidance. US guidance also provides clear images of the sacral hiatus and detects the anatomic variations of the sacrum and sacral hiatus. In the previous studies, approximately 2% to 3% of the studied population had closed sacral canals, thus making “cervical epidural steroid injection” impossible for these subjects [17]. US-guided caudal epidural steroid injections” have been reported to achieve a 100% success rate in identifying the sacral hiatus in patients with radicular pain.

This systematic review has several major limitations. Only eight studies with relatively small sample sizes matched the inclusion criteria and were meta-analyzed. There was heterogeneity in the patient populations, the anatomical localization of back pain, and interventions included in the meta-analysis. Additionally, some studies employed an “ultrasound-assisted” approach, where ultrasound was used to locate the injection site, while others utilized dynamic guidance during the procedure. Given the nature of the intervention, there was a serious risk of bias in the included studies due to the lack of blinding. Finally, while there was no statistically significant difference observed between the two techniques, none of the included studies had a no-intervention arm to assess the effectiveness of the injections for low back pain. Despite these limitations, there was no evidence that US-guided techniques were overall inferior to FL-guided techniques. The advantages of the US include relative simplicity of performance, absence of radiological exposure, and continuous real-time needle guidance. However, while ultrasound guidance can decrease radiation exposure and enhance the visualization of soft tissues and vascular structures, there are a few disadvantages [29]. The quality of images can be impacted by patient morphology and injection location, and the technique is not effective for visualizing axial or spine structures where bone produces an acoustic shadow [32].

## 5. Conclusions

There were no significant differences between US-guided and FL-guided injections in reducing back pain intensity and complications at one month and three months after intervention. However, the model tends to favor the FL-guided injections over the US-guided injections in terms of functionality. Given the limitations of this meta-analysis, future RCTs are warranted to establish solid evidence on relevant outcomes for using these two techniques for back pain management.

## Figures and Tables

**Table 2:** Cochrane risk of bias.

Study	Randomization Process	Deviations from the Intended Interventions	Missing Outcome Data	Measurement of the Outcome	Selection of the Reported Result	Overall Risk of Bias
Hazra et al., 2016 [16]	Some concerns	Low risk	Low risk	Low risk	Low risk	Some concerns
Soneji et al., 2016 [17]	Some concerns	Low risk	Some concerns	Low risk	Low risk	Some concerns
Park et al., 2013 [18]	Some concerns	Some concerns	Some concerns	Low risk	Low risk	High risk
Akkaya et al., 2017 [6]	Some concerns	Low risk	Low risk	Low risk	Low risk	Some concerns
Evansa et al., 2015 [3]	Some concerns	Some concerns	Some concerns	Low risk	Low risk	High risk
Jee et al., 2013 [2]	Some concerns	Some concerns	Some concerns	Low risk	Low risk	High risk
Yang et al., 2016 [7]	Some concerns	Some concerns	Some concerns	Low risk	Low risk	High risk
Jee et al., 2014 [19]	Some concerns	Low risk	Low risk	Low risk	Low risk	Some concerns

**Table 3:** Summary of findings. CI, confidence interval.

Outcomes	Risk Ratio [95% CI]	Standardized Mean Difference [95% CI]	Number of Participants (Studies)	Certainty of the Evidence (GRADE)
Postoperative pain at 1 month	-	0.10 [−0.12, 0.33]	312 (5)	⊕⊕⊕⊕ High
Postoperative functionality (ODI index) at 1 month	-	0.02 [−0.24, 0.28]	232 (4)	⊕⊕⊕⊕ High
Vasovagal reaction	0.96 [0.21, 4.41]	-	240 (4)	⊕⊕⊕◯ Moderate ^a^
Transient headache	1.04 [0.16, 6.87]	-	240 (4)	⊕⊕⊕◯ Moderate ^a^
Facial flushing	0.99 [0.41, 2.36]	-	232 (4)	⊕⊕⊕◯ Moderate ^a^

^a^ Due to inconsistency (wide variance of point estimates) and imprecision (wide confidence interval).

## Data Availability

The data presented in this study are available upon request from the corresponding author.

## Supplementary Materials

The following supporting information can be downloaded at https://www.mdpi.com/article/10.3390/diagnostics13223474/s1. Table S1: Evidence profile.

## References

[1] Chen C.P.C., Tang S.F.T., Hsu T.-C., Tsai W.-C., Liu H.-P., Chen M.J.L., Date E., Lew H.L. (2004). Ultrasound Guidance in Caudal Epidural Needle Placement. Anesthesiology.

[2] Jee H., Lee J.H., Kim J., Park K.D., Lee W.Y., Park Y. (2013). Ultrasound-Guided Selective Nerve Root Block versus Fluoroscopy-Guided Transforaminal Block for the Treatment of Radicular Pain in the Lower Cervical Spine: A Randomized, Blinded, Controlled Study. Skeletal Radiol..

[3] Evansa I., Logina I., Vanags I., Borgeat A. (2015). Ultrasound versus Fluoroscopic-Guided Epidural Steroid Injections in Patients with Degenerative Spinal Diseases: A Randomised Study. Eur. J. Anaesthesiol..

[4] Stitz M.Y., Sommer H.M. (1999). Accuracy of Blind versus Fluoroscopically Guided Caudal Epidural Injection. Spine.

[5] Manchikanti L., Cash K.A., Pampati V., McManus C.D., Damron K.S. (2004). Evaluation of Fluoroscopically Guided Caudal Epidural Injections. Pain Physician.

[6] Akkaya T., Ozkan D., Kertmen H., Sekerci Z. (2017). Caudal Epidural Steroid Injections in Postlaminectomy Patients: Comparison of Ultrasonography and Flouroscopy. Turk. Neurosurg..

[7] Yang G., Liu J., Ma L., Cai Z., Meng C., Qi S., Zhou H. (2016). Ultrasound-Guided Versus Fluoroscopy-Controlled Lumbar Transforaminal Epidural Injections: A Prospective Randomized Clinical Trial. Clin. J. Pain.

[8] Loizides A., Gruber H., Peer S., Galiano K., Bale R., Obernauer J. (2013). Ultrasound Guided versus CT-Controlled Pararadicular Injections in the Lumbar Spine: A Prospective Randomized Clinical Trial. AJNR Am. J. Neuroradiol..

[9] Hashemi M., Dadkhah P., Taheri M., Haji Seyed Abootorabi S.M., Naderi-Nabi B. (2019). Ultrasound-Guided Lumbar Transforaminal Epidural Injections; A Single Center Fluoroscopic Validation Study. Bull. Emerg. Trauma.

[10] Blanchais A., Le Goff B., Guillot P., Berthelot J.-M., Glemarec J., Maugars Y. (2010). Feasibility and Safety of Ultrasound-Guided Caudal Epidural Glucocorticoid Injections. Joint Bone Spine.

[11] Page M.J., McKenzie J.E., Bossuyt P.M., Boutron I., Hoffmann T.C., Mulrow C.D., Shamseer L., Tetzlaff J.M., Akl E.A., Brennan S.E. (2021). The PRISMA 2020 Statement: An Updated Guideline for Reporting Systematic Reviews. BMJ.

[12] Luo D., Wan X., Liu J., Tong T. (2018). Optimally Estimating the Sample Mean from the Sample Size, Median, Mid-Range, and/or Mid-Quartile Range. Stat. Methods Med. Res..

[13] Wan X., Wang W., Liu J., Tong T. (2014). Estimating the Sample Mean and Standard Deviation from the Sample Size, Median, Range and/or Interquartile Range. BMC Med. Res. Methodol..

[14] Sterne J.A.C., Savović J., Page M.J., Elbers R.G., Blencowe N.S., Boutron I., Cates C.J., Cheng H.-Y., Corbett M.S., Eldridge S.M. (2019). RoB 2: A Revised Tool for Assessing Risk of Bias in Randomised Trials. BMJ.

[15] Guyatt G.H., Oxman A.D., Schünemann H.J., Tugwell P., Knottnerus A. (2011). GRADE Guidelines: A New Series of Articles in the Journal of Clinical Epidemiology. J. Clin. Epidemiol..

[16] Hazra A.K., Bhattacharya D., Mukherjee S., Ghosh S., Mitra M., Mandal M. (2016). Ultrasound versus Fluoroscopy-Guided Caudal Epidural Steroid Injection for the Treatment of Chronic Low Back Pain with Radiculopathy: A Randomised, Controlled Clinical Trial. Indian. J. Anaesth..

[17] Soneji N., Bhatia A., Seib R., Tumber P., Dissanayake M., Peng P.W.H. (2016). Comparison of Fluoroscopy and Ultrasound Guidance for Sacroiliac Joint Injection in Patients with Chronic Low Back Pain. Pain Pract..

[18] Park Y., Lee J.-H., Park K.D., Ahn J.K., Park J., Jee H. (2013). Ultrasound-Guided vs. Fluoroscopy-Guided Caudal Epidural Steroid Injection for the Treatment of Unilateral Lower Lumbar Radicular Pain: A Prospective, Randomized, Single-Blind Clinical Study. Am. J. Phys. Med. Rehabil..

[19] Jee H., Lee J.-H., Park K.D., Ahn J., Park Y. (2014). Ultrasound-Guided versus Fluoroscopy-Guided Sacroiliac Joint Intra-Articular Injections in the Noninflammatory Sacroiliac Joint Dysfunction: A Prospective, Randomized, Single-Blinded Study. Arch. Phys. Med. Rehabil..

[20] Kimura R., Yamamoto N., Watanabe J., Ono Y., Hongo M., Miyakoshi N. (2023). Comparative Efficacy of Ultrasound Guidance and Fluoroscopy or Computed Tomography Guidance in Spinal Nerve Injections: A Systematic Review and Meta-Analysis. Eur. Spine J..

[21] Jang J.H., Lee W.Y., Kim J.W., Cho K.R., Nam S.H., Park Y. (2020). Ultrasound-Guided Selective Nerve Root Block versus Fluoroscopy-Guided Interlaminar Epidural Block versus Fluoroscopy-Guided Transforaminal Epidural Block for the Treatment of Radicular Pain in the Lower Cervical Spine: A Retrospective Comparative Study. Pain Res. Manag..

[22] Touboul E., Salomon-Goëb S., Boistelle M., Sobhy Danial J., Deprez V., Goëb V. (2022). Lumbar Zygapophyseal Joints Injections under Ultrasound Guidance an Alternative to Fluoroscopy Guidance in the Management of Low Back Pain. Sci. Rep..

[23] Park K.D., Kim T.K., Lee W.Y., Ahn J., Koh S.H., Park Y. (2015). Ultrasound-Guided Versus Fluoroscopy-Guided Caudal Epidural Steroid Injection for the Treatment of Unilateral Lower Lumbar Radicular Pain: Case-Controlled, Retrospective, Comparative Study. Medicine.

[24] Soto Quijano D.A., Otero Loperena E. (2018). Sacroiliac Joint Interventions. Phys. Med. Rehabil. Clin. N. Am..

[25] Pekkafahli M.Z., Kiralp M.Z., Başekim C.C., Silit E., Mutlu H., Oztürk E., Kizilkaya E., Dursun H. (2003). Sacroiliac Joint Injections Performed with Sonographic Guidance. J. Ultrasound Med..

[26] Klauser A., De Zordo T., Feuchtner G., Sögner P., Schirmer M., Gruber J., Sepp N., Moriggl B. (2008). Feasibility of Ultrasound-Guided Sacroiliac Joint Injection Considering Sonoanatomic Landmarks at Two Different Levels in Cadavers and Patients. Arthritis Rheum..

[27] Hartung W., Ross C.J., Straub R., Feuerbach S., Schölmerich J., Fleck M., Herold T. (2010). Ultrasound-Guided Sacroiliac Joint Injection in Patients with Established Sacroiliitis: Precise IA Injection Verified by MRI Scanning Does Not Predict Clinical Outcome. Rheumatology.

[28] Ashmore Z.M., Bies M.M., Meiling J.B., Moman R.N., Hassett L.C., Hunt C.L., Cohen S.P., Hooten W.M. (2022). Ultrasound-Guided Lumbar Medial Branch Blocks and Intra-Articular Facet Joint Injections: A Systematic Review and Meta-Analysis. Pain Rep..

[29] Hofmeister M., Dowsett L.E., Lorenzetti D.L., Clement F. (2019). Ultrasound- versus Fluoroscopy-Guided Injections in the Lower Back for the Management of Pain: A Systematic Review. Eur. Radiol..

[30] Migliore A., Sorbino A., Bacciu S., Bellelli A., Frediani B., Tormenta S., Pirri C., Foti C. (2020). The Technique of Intradiscal Injection: A Narrative Review. Ther. Clin. Risk Manag..

[31] Wang D. (2018). Image Guidance Technologies for Interventional Pain Procedures: Ultrasound, Fluoroscopy, and CT. Curr. Pain Headache Rep..

[32] Peng P.W.H., Narouze S. (2009). Ultrasound-Guided Interventional Procedures in Pain Medicine: A Review of Anatomy, Sonoanatomy, and Procedures: Part I: Nonaxial Structures. Reg. Anesth. Pain Med..

