# Supplementary material for: Ultrasound-Guided vs. Fluoroscopy-Guided Interventions for Back Pain Management: A Systematic Review and Meta-Analysis of Randomized Controlled Trials
Source: Diagnostics (Basel). 2023 Nov 18;13(22):3474. doi: 10.3390/diagnostics13223474 (PMC10670286; doi:10.3390/diagnostics13223474)
Supplement: Supplementary file 1 [file diagnostics-13-03474-s001.zip › diagnostics-2688527-supplementary.pdf]

Table S1. Evidence profile.

	Postoperative pain at 1 month	Postoperative functionality (ODI index) at 1 month	Vasovagal reaction	Transient headache	Facial flushing
**Risk of bias**	**Not serious**	**Not serious**	**Not serious**	**Not serious**	**Not serious**
Lack of allocation concealment	No	No	No	No	No
Lack of blinding	No	No	No	No	No
Incomplete accounting of patients and outcome events	No	No	No	No	No
Selective outcome reporting	No	No	No	No	No
Other limitations	No	No	No	No	No
**Inconsistency**	**Not serious**	**Not serious**	**Serious**	**Serious**	**Serious**
I^2 (unexplained heterogeneity of results)	No	No	No	No	No
Wide variance of point estimates	No	No	Yes	Yes	Yes
Confidence intervals (CIs) do not overlap	No	No	No	No	No
**Indirectness**	**Not serious**	**Not serious**	**Not serious**	**Not serious**	**Not serious**
Differences in population	No	No	No	No	No
Differences in interventions	No	No	No	No	No
Differences in outcome measures	No	No	No	No	No
Indirect comparisons	No	No	No	No	No
**Imprecision**	**Not serious**	**Not serious**	**Serious**	**Serious**	**Serious**
Few patients	No	No	No	No	No

Wide confidence interval (CI)	No	No	Yes	Yes	Yes
**Upgrading**	**None**	**None**	**None**	**None**	**None**
RR>2 or RR<0.5<br>RR>5 or RR<0.2	No	No	No	No	No
Dose-response gradient	No	No	No	No	No
Effect of plausible residual confounding	No	No	No	No	No
